# Acute toxicity in prostate cancer patients treated with and without image-guided radiotherapy

**DOI:** 10.1186/1748-717X-6-145

**Published:** 2011-10-28

**Authors:** Suki Gill, Jessica Thomas, Chris Fox, Tomas Kron, Aldo Rolfo, Mary Leahy, Sarat Chander, Scott Williams, Keen Hun Tai, Gillian M Duchesne, Farshad Foroudi

**Affiliations:** 1 Department of Radiation Oncology, Peter MacCallum Cancer Centre, Melbourne, Australia; 2 Department of Physical Sciences, Peter MacCallum Cancer Centre, Melbourne, Australia; 3 Radiation Therapy Services, Peter MacCallum Cancer Centre, Melbourne, Australia; 4 Department of Nursing, Peter MacCallum Cancer Centre, Melbourne, Australia; 5 Department of Pathology, University of Melbourne, Melbourne, Australia

**Keywords:** Acute toxicity, image-guided radiotherapy, prostate cancer

## Abstract

**Background:**

Image-guided radiotherapy (IGRT) increases the accuracy of treatment delivery through daily target localisation. We report on toxicity symptoms experienced during radiotherapy treatment, with and without IGRT in prostate cancer patients treated radically.

**Methods:**

Between 2006 and 2009, acute toxicity data for ten symptoms were collected prospectively onto standardized assessment forms. Toxicity was scored during radiotherapy, according to the Common Terminology Criteria Adverse Events V3.0, for 275 prostate cancer patients before and after the implementation of a fiducial marker IGRT program and dose escalation from 74Gy in 37 fractions, to 78Gy in 39 fractions. Margins and planning constraints were maintained the same during the study period. The symptoms scored were urinary frequency, cystitis, bladder spasm, urinary incontinence, urinary retention, diarrhoea, haemorrhoids, proctitis, anal skin discomfort and fatigue. Analysis was conducted for the maximum grade of toxicity and the median number of days from the onset of that toxicity to the end of treatment.

**Results:**

In the IGRT group, 14228 toxicity scores were analysed from 249 patients. In the non-IGRT group, 1893 toxicity scores were analysed from 26 patients. Urinary frequency ≥G3 affected 23% and 7% in the non-IGRT and IGRT group respectively (p = 0.0188). Diarrhoea ≥G2 affected 15% and 3% of patients in the non-IGRT and IGRT groups (p = 0.0174). Fatigue ≥G2 affected 23% and 8% of patients in the non-IGRT and IGRT groups (p = 0.0271). The median number of days with a toxicity was higher for ≥G2 (p = 0.0179) and ≥G3 frequency (p = 0.0027), ≥G2 diarrhoea (p = 0.0033) and ≥G2 fatigue (p = 0.0088) in the non-IGRT group compared to the IGRT group. Other toxicities were not of significant statistical difference.

**Conclusions:**

In this study, prostate cancer patients treated radically with IGRT had less severe urinary frequency, diarrhoea and fatigue during treatment compared to patients treated with non-IGRT. Onset of these symptoms was earlier in the non-IGRT group. IGRT results in less acute toxicity during radiotherapy in prostate cancer.

## Background

Online image-guided radiotherapy (IGRT) in prostate cancer refers to daily pre-treatment imaging and immediate correction for the day to day movement of the target [1,2], caused predominantly by bladder or rectal filling [3]. Schallenkamp et al. reported that between the planning CT and delivery of each fraction of radiotherapy, prostate 3-D displacement ranged from 0.4 to 17 mm with a mean of 5.6 mm [4]. A geographical miss of the prostate will result in increased dose delivered to the adjacent structures.

In a large cohort of patients treated with IGRT for prostate cancer, Lips et al. reported acute grade 2 (G2) genitourinary (GU) toxicity in 47% and G2 gastrointestinal (GI) toxicity in 30% [5]. For comparison, Lips et al. tabulated the acute toxicities from different non-IGRT studies. Acute G2 GU toxicity was seen in 24-49% of patients treated with non-IGRT and acute G2 GI toxicity was seen in 28-57% [5]. In another cohort of 238 patients treated with IGRT, Soete et al. reported a higher rate of 16% with grade 3 (G3) GU and 6% G3 GI toxicity [6]. Conversely, Ghadjar et al. found 56% had G2 GU toxicity but only 3% had G2 GI toxicity [7]. Other studies have reported varying IGRT acute toxicity rates with tomotherapy [8] and conformal radiotherapy [9]. The wide range of GU and GI side effects across studies is due to differences in methodology employed across studies, thus making comparison difficult to interpret. These studies use different radiotherapy planning dose constraints, treatment delivery techniques, target margins, toxicity assessment tools and patient populations across different treatment centres with varying protocols. The exact benefit of IGRT in terms of acute toxicity reduction compared to non-IGRT treated patients has yet to be determined.

In this study, we report the toxicity profile for a large cohort of prostate cancer patients treated with and without IGRT. In 2007, our department implemented an IGRT program for all prostate cancer patients as standard [10]. As quality assurance, we started collecting acute toxicity data prospectively at the end of 2006, before the implementation of IGRT, so that we would have toxicity data on patients before commencing the IGRT program and after. All patients in both groups were treated with the same protocols, target volume expansion margins, planning constraints and underwent the same toxicity assessments.

## Patients and Methods

Between the 22^nd ^of June 2006 and the 24^th ^of June 2009, 291 patients who received radiotherapy for prostate cancer had their toxicity assessed during treatment. Of these, 26 patients were treated with non-IGRT before implementation of the IGRT program and 265 were treated with IGRT.

The 26 non-IGRT patients had pre-treatment orthogonal verification imaging in the first week of radiotherapy which was matched to bony anatomy on digitally reconstructed radiographs (DRRs) from the planning CT scan. The average bony anatomy displacement was calculated and if more than 5 mm on average in the first week then an isocentre move was made for the remainder of treatment. Pre-treatment orthogonal imaging was conducted once a week after the first week to check for setup accuracy during treatment.

The 265 IGRT patients had three cylindrical gold seed fiducial markers measuring 1 mm diameter by 5 mm length inserted into the base, apex and mid prostate one week before the simulation CT scan. These three fiducial markers were visible on the DRRs and also on daily pretreatment orthogonal imaging. Image guided treatment was conducted by image registration of the pretreatment orthogonal imaging with the corresponding DRRs by three dimensional matching of fiducial marker position and translation of the treatment couch to correct for displacement.

All patients followed a bowel emptying and bladder filling protocol prior to simulation and each fraction of treatment as reported previously [11]. Patients were simulated supine with a bolster under knees and foot-stocks fitted onto an immobilisation board (Combifix-Sinmed, Civco, Kalona, IA). The simulation CT was conducted at 3 mm spacing and 3 mm thickness. Prostate, rectum and femoral heads delineation was performed on the planning CT scan. The CTV was defined as the prostate unless there were high risk features present (T3, Gleason ≥8, PSA≥20), in which case the seminal vesicles were included in the CTV. Intermediate risk patients have the base of the seminal vesicles included.

Margins for CTV to PTV expansion were 10 mm cranio-caudal, laterally and anterior, and 7 mm posteriorly. Rectal volumes were contoured as a solid organ from 1.2 cm above the PTV to 1.2 cm below the PTV. Radiotherapy was planned conformally, using 5 or 7 fields. Rectal dose-volume histogram (DVH) constraints were as follows: (i) 50% of rectal volume to receive less than 50 Gy; (ii) 30% of the volume to receive less than 60 Gy; and (iii) 25% of the volume to receive less than 70 Gy. No bladder dose-volume constraints were used in conformal radiotherapy planning. If the patient did not meet rectal dose constraints on the conformal plan, then the bladder was contoured and planning was performed with intensity modulated radiotherapy (IMRT). Hotspots were avoided on the bladder for IMRT. The proportions treated with IMRT in both groups were compared.

All patients were treated on Varian linear accelerators (Varian Medical Systems, Palo Alto, USA). Non-IGRT patients were prescribed 74 Gy in 2 Gy fractions five days a week and IGRT patients were prescribed 78 Gy in 2 Gy fractions five days a week. Although there was a difference in total dose, toxicity was only assessed during treatment and not after the completion of radiotherapy. Therefore the two extra fractions in the IGRT arm would not have impacted on the majority of assessments which were conducted at approximately equal times in both groups.

### Toxicity Data Collection

Patient toxicity was recorded prospectively by nursing staff, radiotherapy staff or medical staff at booked weekly reviews or at additional reviews. Prior to the introduction of toxicity data collection there was a training program conducted by our senior radiotherapy nurse (M.L.). In the non-IGRT group, toxicity data was collected onto standardized paper assessment forms. In the IGRT group, assessments were recorded electronically utilising the same assessment form on Impac Software (IMPAC Medical Systems Inc., Sunnyvale, CA). Each assessment form had ten questions relating to urinary frequency, cystitis, bladder spasm, urinary incontinence, urinary retention, proctitis, anal skin discomfort, diarrhoea, haemorrhoid symptoms, and fatigue. Patients were actively questioned for each of the ten symptoms during each interview. To minimise observer bias, the assessment forms themselves detailed the specifics of each grade of toxicity, so that the assessor could directly compare and choose the most appropriate grade of toxicity for the patient in front of them.

All symptoms were scored according to the Common Terminology Criteria for Adverse Events V3.0 [12]. Details of the data extraction process from Impac onto a Microsoft Access database (Microsoft Corporation, Redmond, WA) have been published by our group [13].

### Statistical Methods

IGRT and non-IGRT patients' T stage, Gleason score and PSA were compared by Wilcoxon rank sum test. History of transurethral resection of the prostate (TURP), neoadjuvant luteinizing hormone releasing hormone (LHRH) agonist, diabetes and hypertension were compared by Fisher's exact test. Frequency of IMRT use between IGRT and non-IGRT groups was tested by Fisher's exact test. Patients with > G1 (greater than grade 1) toxicity at baseline or those with only one day of toxicity information were not included in the analysis for that toxicity as the focus was on analysing change in symptoms occurring during radiotherapy.

### Severity of toxicity

Two methods were used to report toxicity severity. Firstly we tabulated the frequencies of experiencing at least one ≥G2 or ≥G3 toxicity event for each individual toxicity. These frequencies were compared between IGRT and non-IGRT groups using Fisher's exact test.

Secondly, we grouped urinary frequency, cystitis, urinary incontinence, urinary retention and bladder spasm as GU, and proctitis and diarrhoea as GI toxicities, and reported the overall maximum GU toxicity and maximum GI toxicity for all patients. This was to enable the results of our study to be compared to results from other studies, where acute toxicity had been reported in this form.

### Duration of toxicity

Duration of toxicity was measured as the number of days from onset of a G2 or G3 toxicity until the grade of toxicity returned to less than grade 2, or the end of treatment if it did not improve. We assume that toxicity onset is usually gradual therefore the first reported non-zero toxicity is taken to have appeared midway between the day that the toxicity was recorded and the Friday of the previous week. Where a change in toxicity grade occurred, the duration was calculated as the halfway point between change from one grade of toxicity to another.

For each toxicity and for each patient the number of days a patient experienced a ≥G2 toxicity or ≥G3 toxicity was calculated. The Wilcoxon rank sum test was used to test for a difference in the median number of days of each toxicity between the IGRT and non-IGRT groups.

If the toxicity lasted until the end of treatment, then Day 53 is considered the end of the treatment period for IGRT patients. For non-IGRT patients, day 51 is considered the end of the treatment period because IGRT patients receive 39 fractions of 2 Gy and non-IGRT patients receive only 37 fractions of 2 Gy. Therefore for patients who had a ≥G2 toxicity until the end of treatment, the duration of toxicity would automatically be 2 days longer in the IGRT group, because treatment was 2 days longer.

## Results

Of the 265 IGRT and 26 non-IGRT patients, 16 patients were excluded from the IGRT group because they only had one toxicity assessment during treatment. A further 15 toxicities were excluded from the IGRT group and 3 toxicities were excluded from the non-IGRT group because these toxicities were > G1 at baseline. In total, after exclusions, the IGRT group had 14228 toxicity assessments analysed from 1432 assessment days from 249 patients and the non-IGRT group had 1893 toxicity assessment analysed from 192 assessment days from 26 patients.

Patient baseline characteristics are tabulated in table 1. The difference between T stage, Gleason score and PSA were not statistically significant by Wilcoxon rank sum test (p = 0.8987, p = 0.5782 and p = 0.661 respectively). History of TURP, neoadjuvant LHRH agonist, diabetes and hypertension were not significantly different between IGRT and non-IGRT patients by Fisher's exact test (p = .0.8043, 0.5407, 0.4656, 0.6827 respectively).

**Table 1:** Comparison of patient baseline characteristics in the IGRT and non-IGRT groups

T stage	IGRT	Non-IGRT	P value
1	78(30%)	9(35%)	
	
2	137(52%)	13(50%)	
	
3a	40(15%)	3(12%)	
	
3b	6(2%)	0(0%)	
	
4	0(0%)	1(4%)	
	
Not available	4(1%)	0	

Total	265	26	0.8987

Gleason			
	
≤ 6	61(23%)	9(35%)	
	
7	147(55%)	11(42%)	
	
8	31(12%)	2(8%)	
	
9	21(8%)	3(12%)	
	
10	1(0.4%)	1(4%)	
	
Not available	4(1%)	0	

Total	265	26	0.5782

PSA (ng/ml)			
	
< 4	13(5%)	0(0%)	
	
4-10	126(48%)	14(54%)	
	
10.1-20	85(32%)	8(31%)	
	
> 20	37(14%)	4(15%)	
	
Not available	4(1%)	0	

Total	265	26	0.661

Prior TURP	56 (21%)	6 (23%)	0.8043

Neoadjuvant LHRH agonist	131 (50%)	11 (42%)	0.5407

Diabetes	61 (23%)	4 (15%)	0.4656

Hypertension	145 (55%)	13 (50%)	0.6827

There were 12% of patients who were treated with IMRT in the non-IGRT arm and 4% in the IGRT arm. This difference was not statistically significantly by Fisher's exact test (p value of 0.191).

Table 2 shows the frequency ≥ G2 and ≥ G3 for all ten toxicities. ≥ G3 urinary frequency, ≥ G2 diarrhoea and ≥ G2 fatigue were significantly more common in the non-IGRT group than the IGRT group. ≥ G3 urinary frequency was three times more common in the non-IGRT group compared to the IGRT group (23% vs. 7% p = 0.0188). Urinary frequency and cystitis were the most common toxicities and ≥ G3 events only occurred for urinary frequency and cystitis. There was no difference for ≥ G2 urinary frequency and cystitis between the groups. Of the rectal symptoms, diarrhoea (15% vs. 3% p = 0.0174) was more common in the non-IGRT groups than the IGRT group. Fatigue ≥ G2 was also three times more common in the non-IGRT group compared to the IGRT group (23% vs. 8% p = 0.0271).

**Table 2:** Fisher's exact test comparing the difference in proportions of patients experiencing at least one toxicity event in IGRT and non-IGRT groups

Toxicity	% ≥ grade 2 IGRT non-IGRT p-value			% ≥ grade 3 IGRT non-IGRT p-value		
Urinary frequency	35	52	0.1144	7	23	0.0188

Cystitis	47	42	0.6857	1	4	0.3243

Bladder spasm	1	0	1	0	0	NE*

Urinary incontinence	3	0	1	0	0	NE

Urinary retention	7	0	0.3825	0	0	NE

Proctitis	6	15	0.0862	0	0	NE

Anal skin	8	8	1	0	0	NE

Diarrhoea	3	15	0.0174	0	0	NE

Haemorrhoid symptoms	4	8	0.3097	0	0	NE

Fatigue	8	23	0.0271	0	0	NE

*NE = No grade 3 events.

We grouped all urinary symptoms as GU toxicities, and all rectal symptoms as GI toxicities. Table 3 shows the grouped frequencies of GI and GU toxicities for IGRT and non-IGRT. Overall, GU toxicities were more common that GI toxicities, and also more severe. Most patients experienced a GU toxicity during treatment, 93% for IGRT and 96% for non-IGRT. Although IGRT patients had a higher rate of G2 GU toxicities (54% vs. 38%), this group had relatively less G3 GU toxicities (9% vs. 23%) compared to the non-IGRT group. As more severe toxicities were less common in the IGRT group, there were more patients who experienced less severe toxicity.

**Table 3:** Overall maximum toxicity during treatment: genitourinary (GU) and gastrointestinal (GI)

	GU toxicity		GI toxicity	
**Grade of toxicity**	**Non-IGRT**	**IGRT**	**Non-IGRT**	**IGRT**

Grade 0	1(4%)	19(7%)	12(46%)	115(42%)

Grade 1	9(35%)	80(30%)	9(35%)	132(49%)

Grade 2	10(38%)	145(54%)	5(19%)	23(9%)

Grade 3	6(23%)	23(9%)	0	0

Grade 4	0	0	0	0

There were no grade 3 or 4 GI toxicities. G2 GI toxicity was half as common in the IGRT group compared to the non-IGRT group (9% vs. 19%). Proportionally there were more patients with G1 GI toxicities in the IGRT group, compared to the non-IGRT group, because of less patients in the IGRT group with more severe toxicity.

Table 4 shows the median number of days and interquartile ranges for toxicities of ≥ G2; only considering patients who had the toxicity. The duration of treatment was longer for the IGRT group compared to the non-IGRT group by 2 days. Despite this, patients in the IGRT group had a shorter duration of toxicity overall compared to patients in the non-IGRT group as shown in table 4. The median number of days with a ≥ G2 toxicity was significantly shorter by Wilcoxon rank sum test for ≥ G2 (p = 0.0179) and ≥ G3 frequency (p = 0.0027), ≥ G2 diarrhoea (p = 0.0033) and ≥ G2 fatigue (p = 0.0088) in favour of the IGRT group. This result means that these symptoms occurred later in the treatment course for IGRT patients compared to non-IGRT patients.

**Table 4:** Medians number of days and interquartile ranges for toxicities of ≥ G2

Toxicity	IGRT		Non-IGRT		P value (Wilcoxon Rank Sum test)
		
	Median	Interquartile range	Median	Interquartile range	
Urinary frequency	14.5	10 - 26.5	28	23.4 - 32.9	0.0179

Cystitis	15	8.5 - 27	24.5	19.3 - 31.3	0.7603

Bladder spasm	8.8	8.1 - 9.4	No events	-	0.6566

Urinary incontinence	10.5	7.8 - 15.3	No events	-	0.3919

Urinary retention	8.5	6 - 15.5	No events	-	0.1746

Proctitis	10.5	7.8 - 18	20.8	11.5 - 28.4	0.0616

Anal skin	8.8	6.9 - 18.3	23.3	20.9 - 25.6	0.9308

Diarrhoea	10.3	4.8 - 10.6	9.3	7 - 12.9	0.0033

Haemorrhoid symptoms	15.5	8.6 - 25.6	26	17 - 35	0.3583

Fatigue	10.8	7.4 - 14	18.8	15.1 - 22	0.0088

## Discussion

In our study, urinary frequency, diarrhoea and fatigue occurred less in patients treated with IGRT than in patients treated with non-IGRT. The proportion of benefit appeared to be approximately 2/3 less for all three toxicities. We would expect rectal and bladder side effects to be less with IGRT compared to non-IGRT because IGRT not only reduces the chance of geographical miss of the target but also reduces inadvertent dose deposition in the adjacent normal tissues [14]. Fatigue is a systemic effect but it may be associated with urinary frequency or diarrhoea because of loss of sleep from nocturia or depletion of electrolytes caused by severe diarrhoea. As patients with a baseline toxicity > G1 were excluded from the analysis for that toxicity, it is likely that the maximal toxicity experienced was caused by radiotherapy treatment, as the symptom or symptoms appeared during treatment. The time related analysis and severity analysis were done separately and both were independently significant in favour of IGRT making it less likely that the benefit seen occurred by chance. Organ toxicity is a function of the dose received [15], and the volume of tissue irradiated [16]. Considering that side effects are dose related, our data suggests that organs at risk are getting less dose during treatment with IGRT compared to non-IGRT in this study.

Table 5 compares the results from our study with acute toxicity results from other studies employing IGRT for prostate cancer. As mentioned in the introduction, it is not fully applicable to compare toxicity results across studies, because of the differences in methodology and protocols used in these studies. However as illustrated, the results in the present study are not vastly dissimilar from results in other non-randomized studies employing IGRT, whereby G3 and G4 toxicities are uncommon.

**Table 5:** Comparison to results from other studies using IGRT for prostate cancer

Study	Method	Acute toxicity GU by grade (%)			Acute toxicity GI by grade (%)		
		
		2	3	4	2	3	4
Present study (IGRT) (n = 249)	96% conformal RT fiducials 78 Gy	54	9	0	9	0	0

Lips et al. (5) (n = 331)	IMRT fiducials 76 Gy	47	3	0	30	0	0

Soete et al. (6) (n = 238)	IG Arc therapy	37	16	0	19	6	0

Ghadjar et al. (7) (n = 39)	IMRT fiducials 80 Gy	56	8	0	3	0	0

Cheng et al. (8) (n = 76)	Tomotherapy 78.9 Gy	38	0	0	25	0	0

Martin et al. (9) (n = 259)	87% conformal RT fiducials 79.8 Gy	33	0	0	10	0	0

The largest limitation of our study is that it is not a randomized study. We have tried to limit the confounding factors by demonstrating that the patients were not preselected, and that the T stage, Gleason score, PSA, history of TURP, LHRH agonist, diabetes and hypertension were identical between groups. Although there was a difference in numbers of patients treated with IMRT and non-IMRT, the difference was not statistically significant. In addition, the use of IMRT did not change the dose constraints for organs at risk, and therefore similar doses were allowed to organs at risk with IMRT and without IMRT in this study and no patient would have received grossly outlying organ at risk doses. The second limitation is that there was a substantially smaller number of patients in the non-IGRT group (n = 26) compared to the IGRT group (n = 249). The effect of a small sample size is to reduce the power to detect a difference in toxicity between the two groups, especially for toxicities that occurred less frequently. For example, proctitis and flare of haemorrhoids were also more common in the non-IGRT group compared to the IGRT group, although the difference did not reach statistical significance. If we had more patients in the non-IGRT group, we may have had a greater ability to detect a difference in toxicity level for the less common acute toxicities of radiotherapy.

Several randomised controlled trials and one meta-analysis have demonstrated better biochemical control for dose escalation in prostate cancer radiotherapy [17-21]. However, toxicity is a serious concern in prostate cancer patients receiving dose escalated radiotherapy despite better biochemical control. The lower toxicity for IGRT in our study was found despite the higher total dose in the IGRT arm. The two extra fractions at the end of treatment are more likely to have an effect on late reactions. In dose escalation studies with total dose of 74 Gy to 79.2 Gy, 17-33% of patients had a ≥ G2 late GI toxicity and 11-30% had a ≥ G2 late GU toxicity [17-21]. Several studies have shown that acute toxicity is predictive of late toxicity in prostate cancer patients undergoing radiotherapy [22-25]. In a Trans-Tasman Radiation Oncology Group (TROG) study, ≥ G2 acute toxicity was associated with a greater than threefold risk of the subsequent development of late toxicity [22]. Of patients developing late toxicity, 79% had acute toxicity of ≥ G2. This is not surprising, as the side effects experienced acutely are a function of dose and inherent patient sensitivity and the side effects experienced later on are also a function of dose and sensitivity. In a Dutch study, 75% of men given a choice between two doses of radiotherapy for prostate cancer chose to have the lower dose with the lower cure rate over bladder and bowel treatment related side effects [26]. IGRT is one method which may possibly reduce toxicity whilst maintaining dose escalation.

## Conclusion

In the context of a dose escalation in the IGRT group from 74Gy to 78Gy, compared to non-IGRT, prostate cancer patients treated radically with IGRT had lesser diarrhoea, urinary frequency and lethargy during radiotherapy despite the same CTV to PTV margin in both groups. IGRT should be the standard of practice in dose-escalated radiotherapy.

## Competing interests

The authors declare that they have no competing interests.

## Authors' contributions

SG designed the study, analysed the data and prepared the manuscript. CF extracted, and sorted the data. JT conducted all statistical analyses. AR and ML coordinated training of staff for data collection. SC, SW, KHT, GMD and FF participated in the treatment of the patient cohort described, designed the study and assisted with preparation of the manuscript. All authors read and approved the final manuscript.
